# Quantitative alignment parameter estimation for analyzing X-ray photoelectron spectra

**DOI:** 10.1107/S1600577523004150

**Published:** 2023-06-16

**Authors:** Matthew Ozon, Konstantin Tumashevich, Nønne L. Prisle

**Affiliations:** a Center for Atmospheric Research, PO BOX 4500, University of Oulu, Finland; ESRF – The European Synchrotron, France

**Keywords:** X-ray photoelectron spectroscopy, experimental alignment parameter, measurement model, liquid jet, quantitative data inversion

## Abstract

The interpretation of X-ray photoelectron spectroscopy (XPS) data relies on measurement models that depend on several parameters, including the photoelectron attenuation length and X-ray photon flux. However, some of these parameters are not known, because they are not or cannot be measured. The unknown geometrical parameters can be lumped together in a multiplicative factor, the alignment parameter. This parameter characterizes the ability of the exciting light to interact with the sample. Unfortunately, the absolute value of the alignment parameter cannot be measured directly, in part because it depends on the measurement model. Instead, a proxy for the experimental alignment is often estimated, which is closely related to the alignment parameter. Here, a method for estimating the absolute value of the alignment parameter based on the raw XPS spectra (*i.e.* non-processed photoelectron counts), the geometry of the sample and the photoelectron attenuation length is presented. The proposed parameter estimation method enables the quantitative analysis of XPS spectra using a simplified measurement model. All computations can be executed within the open and free Julia language framework *PROPHESY*. To demonstrate feasibility, the alignment parameter estimation method is first tested on simulated data with known acquisition parameters. The method is then applied to experimental XPS data and a strong correlation between the estimated alignment parameter and the typically used alignment proxy is shown.

## Abstract 2

A model of an X-ray photoelectron spectroscopy experiment accounting for photon beam asperities, sample geometry and kinetic energy analyzer is introduced. This model is related via the alignment parameter to a simple model commonly used for data interpretation. An alignment parameter estimation method is introduced and tested with simulated and experimental data.

## 1. Introduction

X-ray photoelectron spectroscopy (XPS) is an agile chemical and structure analysis technique which is based on the characterization of the energies of electrons emitted from a substance due to excitation with X-ray photons (Watts, 1994 ▸). Due to the high chemical selectivity and surface sensitivity, the method has become widespread in surface science (Hüfner, 1995 ▸). We here consider the case of XPS applied to a liquid microjet (LJ) sample (Winter & Faubel, 2006 ▸). During LJ XPS experiments, the sample is injected through a nozzle as a high-speed jet into the measurement chamber and ionized by the photon beam, leading to emission of photoelectrons in accordance with the photoelectric effect. Spectra are recorded using an electron analyzer to count photoelectrons emitted from ionized core-level orbitals across a range of kinetic energies (Ottosson *et al.*, 2010 ▸; Prisle *et al.*, 2012 ▸). Collected XPS spectra consist of peaks, corresponding to different chemical species or chemical environment in the sample and their chemical environment. XPS is a powerful technique for studying composition and other properties of interfaces, which has recently been successfully applied to aqueous samples with immediate atmospheric relevance (Prisle *et al.*, 2012 ▸; Walz *et al.*, 2015 ▸, 2016 ▸; Werner *et al.*, 2014 ▸, 2018 ▸; Öhrwall *et al.*, 2015 ▸). These studies have provided crucial new insights to advance the understanding of key processes in atmospheric aqueous systems, but are currently hampered because of limited ability to retrieve quantitative information of aqueous interfacial properties from experimental XPS data.

For absolute quantitative analysis of experimental data, all parameters comprising the acquisition model must be determined. In the case of XPS spectra, the measurement model consists of three components: (1) the light, (2) the sample and (3) the measurement device, *i.e.* the electron kinetic energy analyzer. When XPS is combined with synchrotron light, the beam parameters are well defined but may vary between different facilities and beamlines (Fedoseenko *et al.*, 2003 ▸; Petrova *et al.*, 2019 ▸; Kachel, 2016 ▸). Considerable effort has been dedicated to systematically characterize kinetic energy analyzers using an overall parameter to describe the intensity response function of the device (Seah, 1990 ▸, 1993 ▸; Wicks & Ingle, 2009 ▸; Guilet *et al.*, 2022 ▸). The calibration of instruments is a crucial step for each experiment to minimize quantification errors (Roy & Tremblay, 1990 ▸; Seah, 1995 ▸; Dupuy *et al.*, 2021 ▸).

The remaining component, that is the sample, in the XPS acquisition model is characterized by several parameters describing its interaction with the exciting light (*e.g.* photoionization cross section) and emitted electron (*e.g.* attenuation length), as well as its geometry (*e.g.* cylinder). The probability that the exciting light effectively interacts with the probed target in the sample can be summarized by the average photon density with respect to a probability that depends on the density of substance in the sample. This quantity is referred to as the alignment parameter (AP). In the XPS measurement model, the alignment parameter is a dimensioned multiplicative factor akin to a surface density, which depends on the geometry of the sample (Ottosson *et al.*, 2010 ▸), the shape of the photon beam, and the overlap between the beam and the sample. This parameter originates from the simplification of the measurement model used to describe XPS data acquisition. In the papers by Dupuy *et al.* (2021 ▸) and Ottosson *et al.* (2010 ▸) the AP appears in the measurement model used for data interpretation as a proportionality constant with roots in geometry. In these works, the AP is a constant of the experiment that is determined by the geometry of the sample and the arrangement of the sample relative to the spectrometer. However, these works do not offer mathematical definitions or numerical methods to compute or estimate the value of this parameter. Typically, XPS data analysis is based on relative quantities, such as the spectral peak area ratio. When the AP is assumed to be constant for a given experimental setup, using relative quantities seems to cancel out the AP. However, using peak area ratios by definition eliminates one spectrum, which is not desirable in the case of highly limited available data. Therefore, we here present a method for estimating the AP which allows the data analysis to be carried out in absolute terms while also avoiding the loss of one spectrum for relative analysis. In addition to geometry information, the AP also indicates the probability of interaction between the photon beam and target orbital, which is another characteristic of the sample. The purpose of this work is to establish a rigorous definition of the AP, from the assumptions leading to its definition to the practical implications of the defined parameter.

When the LJ comprises an aqueous solution, water is present both in liquid and vapor form, even if experiments are carried out at very low overall pressures (Winter & Faubel, 2006 ▸; Öhrwall *et al.*, 2015 ▸). Therefore, two distinct peaks, one for each phase, are often present in the recorded water O 1*s* spectra. All other things being equal, the relative intensities (peak areas) of the gas peak and the liquid peak depend on the overlap between the X-ray beam and the LJ sample. For instance, if the photon beam targets a region near the LJ without including the liquid phase, no peak corresponding to the liquid will appear in the spectra. On the other hand, if the beam completely illuminates the LJ sample, the photoelectron signal will show peaks for both the liquid and gas phases. The beam spot is typically of the same size or larger (25–100 µm) (Zhu *et al.*, 2021 ▸; Chernenko *et al.*, 2021 ▸) than the LJ sample diameter (20–40 µm). Therefore, the gas peak is always present in the spectrum (Brown *et al.*, 2013*a* ▸; Perrine *et al.*, 2014 ▸; Prisle *et al.*, 2012 ▸; Werner *et al.*, 2014 ▸; Öhrwall *et al.*, 2015 ▸).

For robust results, target and reference signals for the peak area ratio should be measured within a relatively close period of time (to minimize possible changes in the actual alignment between these measurements), from the same solution, and at the same photoelectron kinetic energy (to ensure comparable attenuation with depth). If these conditions are met, the ratio between the target and water O 1*s* peak intensity ratios cancels out the experimental alignment parameter and all target species concentrations are effectively comparable in terms of their concentration in water. However, the use of a data point to cancel out the alignment parameter means the loss of that point for the analysis of the physics of the system under investigation.

**Figure 1:**
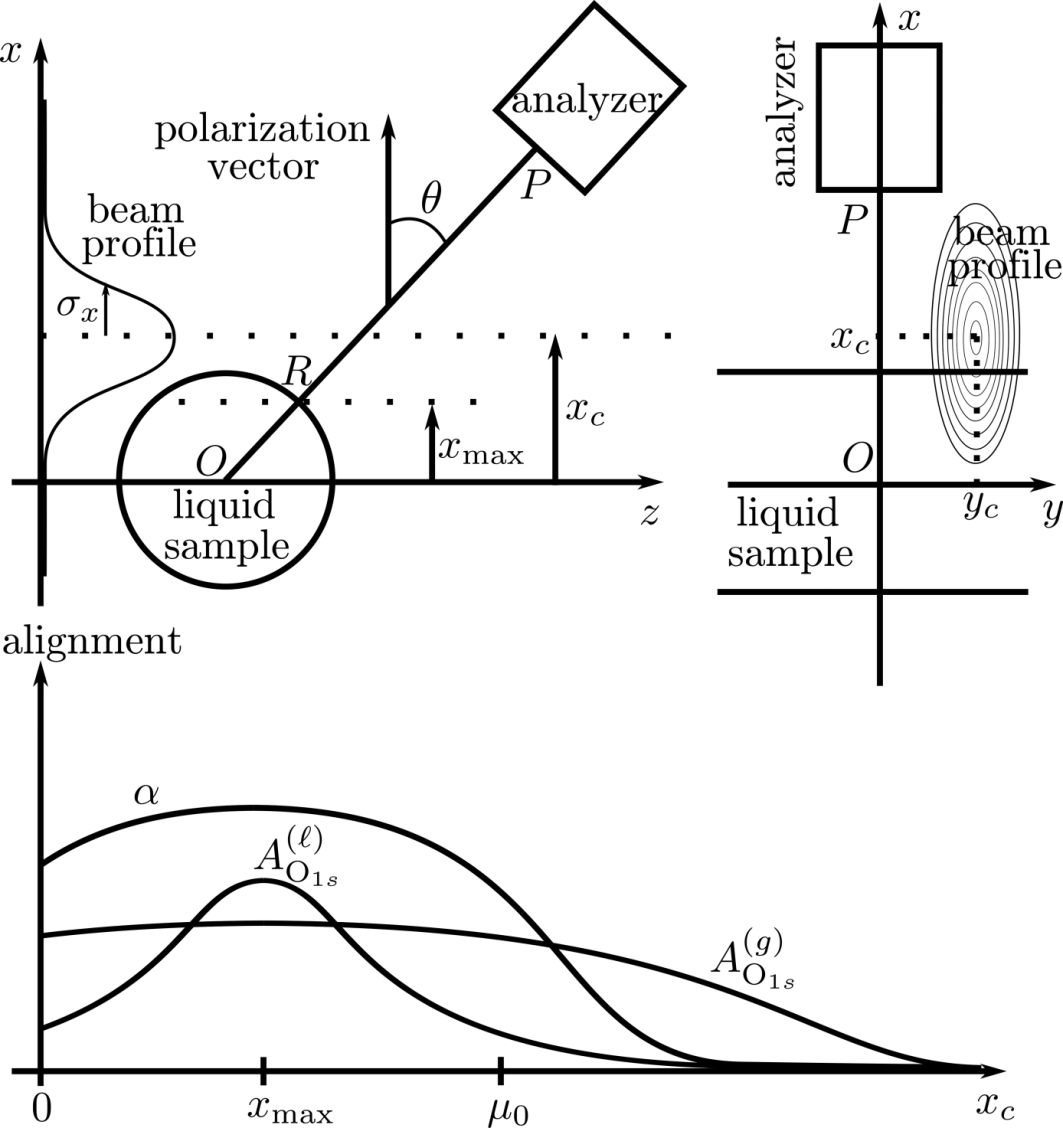
Sketch of the cross section of the acquisition setup in the plane *xOz* with measurement angle θ with respect to the polarization vector. The beam profile is off center by a distance *x*_c_ along the *x*-axis. The O 1*s* peak areas (liquid and gas) and the alignment parameter are plotted against the offset *x*_c_.

The alignment parameter and the peak areas share the same variation pattern with respect to the off-center distance *x*_c_ [m], the minimal distance between the maximum of the beam profile and the symmetry axis of the LJ sample, as schematically illustrated in Fig. 1 ▸. The off-center distance *x*_c_ is not determined directly; instead, it can be estimated by monitoring both the total signal intensity and the dimensionless water O 1*s* liquid-to-gas peak area ratio (LGPAR). The LJ is moved towards the supposed center of the beam spot, after which the relative position is adjusted to obtain the highest possible signal intensity for the liquid peak (Mudryk *et al.*, 2020 ▸). However, the jet alignment may fluctuate due to, for example, formation of ice needles, changes in the efficiency of pumps, nozzle clogging, which can then introduce errors into the data analysis (Ali *et al.*, 2019 ▸; Brown *et al.*, 2013*b* ▸). Therefore, obtaining a stable experimental alignment and robust estimate of the alignment parameter is challenging in particular for LJ and other dynamic samples.

We here present a numerical method for alignment parameter estimation (APE), based on the measured spectra, the geometry of the sample and the attenuation length of the photoelectron signal in the sample, and utilizing the noise in the measured photoelectron spectra. The estimated values of the alignment parameter [from equation (9)] are compared with experimentally determined values of the water O 1*s* LGPAR proxy and values for the theoretical alignment parameter obtained for simulated data [using equation (7)]. The method is here applied to the case of LJ samples, but can be expanded to apply to any liquid or solid samples with known geometry.

## 2. Model

### 2.1. Detailed model

We first devise a detailed model that describes the data collected during XPS experiments and requires knowledge of all relevant experimental parameters. We use the following model for the photoelectron flux of interest 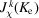 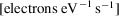 from the target orbital χ with kinetic energy *K*_e_ [eV] generated by irradiating the sample with photons with energy centered around *h*ν_*k*_ [eV] (Fadley, 1978 ▸; Paynter, 1981 ▸),

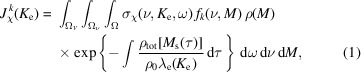

where Ω_ν_ is the spectral integration domain of the photon energy, Ω_*V*_ is the spatial integration domain covering the sample, and Ω is the angular integration domain covering the spectrometer aperture. This model takes into account (1) the light source imperfections through the density function *f*_*k*_(ν, *M*) [photon m^−2^ eV^−1^ s^−1^], (2) the photoionization cross-section spectral and angular density 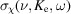 [m^2^ eV^−1^ sterad^−1^] of the target orbital χ, and (3) the exponential attenuation of the emitted signal due to collisions along the path (τ, *M*_s_(τ)) from the point *M* to the aperture of the analyzer in *P*. The concentrations ρ(*M*) [m^−3^] and ρ_tot_(*M*) [m^−3^] at point *M* are those of the species of interest and of all chemical species in the sample, respectively, and λ_e_(*K*_e_) [m] is the photoelectron attenuation length in the sample. In this model, all the quantities are assumed to be either time invariant or represent the time-averaged values. If the time fluctuation of the photon beam *f*_*k*_ and the concentration ρ are stochastically independent from each other, then the time average of equation (1) can be obtained by substituting the time-varying quantities *f*_*k*_ and ρ with their corresponding time-averaged values.

The total photoelectron flux consists of both the target and background signals; however, the latter component is commonly not of interest for data interpretation. The background signal nevertheless contributes to determining the noise level in the measured data and should therefore be represented in the acquisition model. We here define the background electron flux 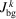 [eV^−1^ s^−1^] as a function to be fitted from experimental spectra, depending on the photon energy *h*ν_*k*_ and the kinetic energy *K*_e_ of the photoelectrons. Contrary to the target signal of interest 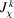 , the background 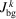 is formed by electrons emitted by multiple processes and potentially not from the interaction with X-ray photons. For instance, a photoelectron contributing to the background signal may come from a valence orbital (rather than a core-level) that underwent inelastic collisions (Stevie & Donley, 2020 ▸; Hesse & Denecke, 2011 ▸).

The overall photoelectron flux creates the signal measured by the kinetic energy analyzer divided into *N*_*k*_ channels spread across the energy range 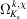 = 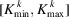 [eV]. The analyzer is a complex system that can be modeled (as a first-order approximation) by *N*_*k*_ efficiency functions that account for phenomena such as the kinetic energy analyzer bandwidth (Seah, 1995 ▸). The expected measured signal in the ℓth channel, a number or intensity of electrons, is well approximated by a convolution of the input signal with an efficiency function (Popović *et al.*, 2017 ▸; Paolini & Theodoridis, 1967 ▸), 

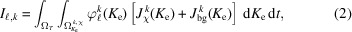

where 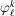 is the efficiency function of the ℓth measurement channel which is centered at the kinetic energy 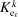 [eV], with bandwidth 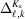 [eV] and intensity response function 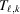 [a.u.]. The discrete kinetic energy values 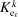 = 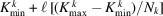 cover a range of energy that depends on the target species which is adjusted according to experimental conditions, *i.e.* 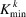 , 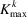 and *N*_*k*_ are tuned to best sample the targeted orbital (Baer, 2020 ▸). The intensity response function of the instrument 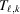 includes all instrumental parameters of the XPS receiver, specifically the transmissions of the entrance lens, electrostatic lens, hemispherical energy analyzer, the exit lens, the efficiency of the detector, and the contribution of the electronics (Seah, 1990 ▸; Guilet *et al.*, 2022 ▸; Wicks & Ingle, 2009 ▸; Popović *et al.*, 2017 ▸; Trigueiro *et al.*, 2018 ▸), and does not vary significantly over 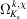 for a fixed pass energy. The instrumental response function can be measured experimentally when calibrating the analyzer (Seah, 1990 ▸; Guilet *et al.*, 2022 ▸). Despite its complexity, 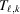 can be approximated for a given device by a polynomial function in the ratio of the pass energy and the kinetic energy of the emitted electrons (Wicks & Ingle, 2009 ▸). The analyzer efficiency functions 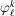 can be approximated by the Dirac δ function with gain 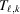 if the bandwidth 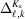 is small compared with the extent of the cross-section density, *e.g.* 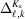 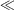 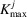 − 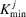 . The time integral signifies that electrons are counted over a finite time interval Ω_*T*_ of length 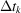 [s]. Here, we consider only the steady state case, so that the time integral in equation (2) can be substituted by a multiplication with 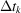 .

The measurement of XPS spectra is a stochastic counting process of (multiplied) photoelectrons. For charged coupled devices (CCDs) (Healey & Kondepudy, 1994 ▸; Konnik & Welsh, 2014 ▸) or a channel electron multiplier (CEM) (Seah, 1990 ▸; Choi & Kim, 2000 ▸) it can be modeled by a Poisson (shot) noise, where the parameter in the form of the expected signal *I*_ℓ,*k*_ is perturbed by the dark current proportional to the integration time 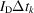 [electrons] as well as by the read noise *N*_read_ [electrons]. For the sake of clarity, we here neglect both the dark current noise, which fades with decreasing temperature, and the reading noise. Hence, the output of the analyzer is given by 

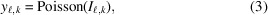

where the expected electron count *I*_ℓ,*k*_ is also the variance, hence the signal-to-noise ratio (SNR) is simply (*I*_ℓ,*k*_)^1/2^. As a consequence, the longer the integration time, the higher the SNR [∝ (Δ*t*_*k*_)^1/2^].

Even though the background signal does not play a role in the definition of the alignment parameter equation (7), it contributes to the noise level in the data since *I*_ℓ,*k*_ is the sum of the contribution of the signal of interest and the background. Because the noise is the foundation of the estimation method, equation (9), the background signal must be accounted for in the measurement model.

### 2.2. Simplified model

In practice, the parameters of the detailed model described by equations (1), (2) and (3) are not all readily measurable within the time frame of an experiment. In particular, the acquisition geometry parameters, *e.g.* the photon beam intensity profile in the frame of reference of the sample, play a crucial role, but they are typically not monitored or recorded during experiments. To reduce the number of unknown parameters in the model, the geometry parameters can be lumped together in a single multiplicative factor under simplifying assumptions.

The photon (spatial and spectral) density *f*_*k*_ of the beam is approximated by 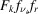 where *F*_*k*_ is the total photon flux [photons s^−1^], and 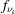 [eV^−1^] and *f*_*r*_ [m^−2^] are the spectra and spatial density, respectively. The beam profile *f*_*r*_ can be measured by positioning a camera at the intersection along the beam. For simulations of spectra, the beam profile can be approximated by a Gaussian function (Fedoseenko *et al.*, 2003 ▸) with center (*x*_c_, *y*_c_) [µm] and spatial spread (σ_*x*_, σ_*y*_) [µm] as described in Section 3.1. The frame is chosen so that the polarization vector of the incident photon beam is along the *x*-axis, the *z*-axis is along the propagation vector of the light, and the *y*-axis coincides with the symmetry axis of the sample cylinder. The spatial spread of the beam is well characterized; however, the relative offset between the center of the sample and the center of the beam profile is not known. Therefore, the photon flux density is approximated as a source uniformly illuminating the sample. In a similar approach, the spectral density 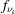 of the photon source can be approximated by a Gaussian centered at *h*ν_*k*_ [eV] with bandwidth 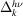 [eV], but it is approximated by a monochromatic distribution with density concentrated at *h*ν_*k*_ [eV].

The total concentration profile ρ_tot_ = ρ_tot_(*M*) of the sample is not well defined at the liquid–vapor interface, but is a smoothly varying function with radial distance *r* = *∥OM∥*, here approximated by a step function equal to the total bulk concentration ρ_0_ within the sample and vanishing outside. The resulting function 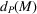 = 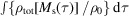 is the distance traveled by emitted photoelectrons in the sharp-edge sample from the point *M* in the direction of the analyzer *P*.

In equation (1), the photoionization cross-section density depends on the spherical angle pair ω = (θ, φ), where the polar angle θ is defined with respect to the polar axis, *i.e.* the polarization vector of the light (see Fig. 1 ▸), such that the spread of the polarization (Petrova *et al.*, 2019 ▸) direction is neglected. The azimuthal angle φ does not carry any information on the cross section of a given orbital for photoionization, because the emitted photoelectrons may originate from any of the magnetic quantum numbers, thereby effectively averaging out any variation with respect to the azimuth (Bethe & Salpeter, 2012 ▸). The remaining angular dependence can be approximated by 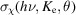 = 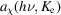 + 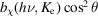 for the case of the dipole approximation for a central potential. The parameters 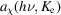 and 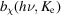 can be estimated from spherical harmonics integration [see equation (72.4) of Bethe & Salpeter (2012 ▸)]. Finally, we use the integrated cross section over the emission angles covering the analyzer aperture to obtain the spectral density of the photoionization cross section as 

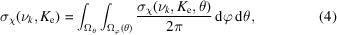

where Ω_θ_ and Ω_φ_ describe the solid angle integration domain Ω covering the analyzer aperture around the direction ω_0_ = (θ_0_, φ_0_). For a small aperture α_ω_, 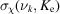 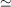 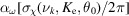 .

Over the kinetic energy interval 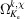 covered by the kinetic energy analyzer (over the domain of the cross-section density of the target species) for photon energy *h*ν_*k*_, the attenuation length λ_e_(*K*_e_) does not vary significantly in a typical setup, *i.e.* 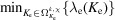 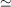 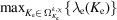 . Hence, we introduce the constant attenuation lengths 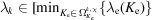 , 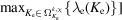 , one for each photon energy*h*ν_*k*_ and target species (dependence not shown for the sake of clarity).

### 2.3. APE method

To bridge between the simplified and detailed models, we now introduce the so-called alignment parameter which is a scaling factor that we denote 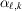 .

We then write the simplified model 

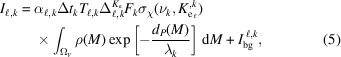

where the expected background signal 

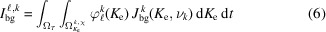

is estimated by a background (or baseline) removal algorithm, such as that described by Baek *et al.* (2015 ▸). With this, the definition of the AP is

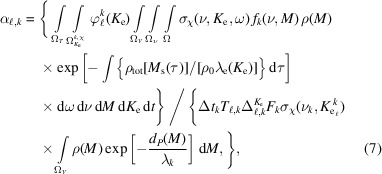

and its dimension is that of a surface density [m^−2^] (effective photon density). The fluctuations in the total photon flux occurring over a short time interval Ω_*T*_, typically a few tens of milliseconds, are smoothed out during the acquisition. The numerical value of the total photon flux, denoted by *F*_*k*_ in equations (5) and (7), is the time average over Ω_*T*_. Therefore, the total photon flux should be recorded in the metadata for each time interval Ω_*T*_. The AP can be broken down into three concepts: (1) an intrinsic property of the substance in the sample (carried by the photoionization cross section), (2) the sample properties (concentrations ρ and ρ_tot_, the attenuation length λ_e_ and the area being illuminated), and (3) the instrumental contributions (spectrometer and light energy spectrum). The instrumental contributions represent how well the photoionization cross-section density can be sampled. With current spectrometer technology, instrumental calibration techniques (Guilet *et al.*, 2022 ▸) and synchrotron light, these effects contribute negligibly to the value of the AP. Hence, the major contribution to the AP comes from the sample properties. The AP is the average value of the photon density with respect to the probability of electron emitters { 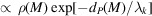 }. Therefore, α represents the average probability density of interaction between the photon beam and the sample which will produce an electron emerging from the sample in the direction of the analyzer. From equation (3), we have readily

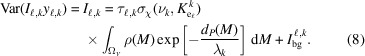

Hence, for each sampled photoelectron kinetic energy 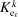 , the parameter 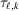 = 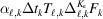 can be estimated by

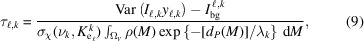

where the target species concentration ρ = ρ(*r*) can be approximated within the sample as the target bulk concentration ρ_B_ [m^−3^] and elsewhere set to 0. The variance of the Poisson distribution is estimated by the realization *y*_ℓ,*k*_, *i.e.* the measurement. The background signal and the cross-section density are presumed known, either computed from first principles or estimated from the data, *e.g.* estimated from a background removal and spectral fitting routine such as *SPANCF* (Kukk *et al.*, 2001 ▸, 2005 ▸). The bias of the estimator is directly related to the bias in the background estimate and in the spectral fits. From the definition equation (7), the values α_ℓ,*k*_ are expected to be almost constant across the kinetic energies 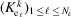 for a given photon energy *h*ν_*k*_. This is because the coarsest approximations in the simplified model equation (5) concern the photon beam profile and the sample geometry, both of which are independent of the kinetic energy. From here, we define the estimator for the expected value of the AP by

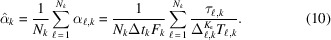

The AP estimator in this formulation accounts for the variations in the intensity response function *T*_ℓ,*k*_ and channel bandwidth 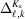 . Note that if a combination of parameters such as α_ℓ,*k*_ and Δ*t*_*k*_ are missing, their product can also be estimated from the samples τ_ℓ,*k*_. In that case, the meaning of the estimation from the numerical samples 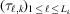 will be different from the estimator defined by equation (10); however, absolute interpretation of data can still be carried out. The product of the alignment parameter and all sample independent parameters (Δ*t*_*k*_, *T*_ℓ,*k*_, 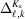 and *F*_*k*_) can be estimated. In Section 4.2, the stochastic samples 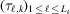 are used for estimation of the product 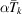 defined in equation (28), where 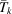 is the average value of the intensity response function over the kinetic energy interval 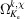 of the acquisition. Consequently, the remaining quantity 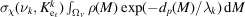 can be evaluated numerically in absolute terms, without involving a spectral peak area ratio. We further notice that from equation (7), all things being equal, the values of α_ℓ,*k*_ should also be independent of the photon energy because of the normalization by all parameters that varies with *h*ν_*k*_, including the attenuation length λ_*k*_. This is illustrated in Fig. 6(*b*) where for each setup the AP is constant across the values of photon energy. From this observation, we define the estimator of the global AP for *K* probed photon energies, 

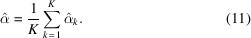

In practice, the measurement model is discretized in space and we denote *H*_*P*_(λ_*k*_) and ρ as the vectors that discretize the spatial integration and the target concentration, so that 

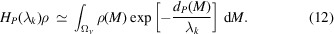

The photoionization cross-section density 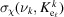 can either be simulated (Toffoli *et al.*, 2007 ▸) or fitted from data. The total atomic photoionization cross section of the target orbital may be taken from established tables (Yeh & Lindau, 1985 ▸). We here consider only fitted cross-section densities.

## 3. Data

The data used here consist of either experimental or simulated XPS spectra. Each spectrum may be recorded for a given photon energy, or set of photon energies, and multiple times in order to increase the data quality (higher SNR). Each chemical state of a target core orbital has a specific spectral signature, which is approximated by one or more peaks. In the case of simulated data, the spectral peaks can be pre-ascribed, whereas for experimental data the number of peaks necessary for describing a given signature is not known *a priori*. Typically, experimental peaks are identified and quantified during spectral fitting. Here, the spectral fitting routine, including background removal, was performed in *IGOR PRO* (Wavemetrics Inc, USA) using the *SPANCF* (Kukk *et al.*, 2001 ▸, 2005 ▸) package. *SPANCF* performs iterative least-squares fitting using the Simplex (Caceci & Cacheris, 1984 ▸) or the Levenberg–Marquardt (Levenberg, 1944 ▸) method. The background for the fit is defined as a simple linear or Shirley (Shirley, 1972 ▸) baseline. The peaks are fitted to the spectrum along with the background in the form of, for example, Gaussian or Lorentzian profiles.

### 3.1. Simulated data

**Figure 2:**
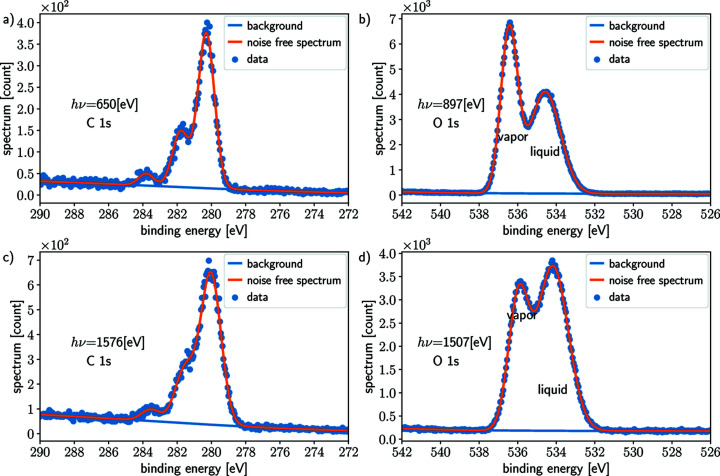
Illustration of the simulation of data using the detailed model described by equations (1), (2) and (3). The simulated C 1*s* for 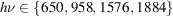 eV and O 1*s* for *h*ν ∈ {897, 1507} eV data are generated using a sharp edge volume approximation and a smooth edged profile. The C 1*s* spectra in panels (*a*) and (*c*) exhibit three peaks, χ_1_, χ_2_ and χ_3_, one for each chemical state of carbon in the simulated system, characterized by the mode and width determined by the polynomial models, equations (19)–(24). The water O 1*s* spectra are simulated with two peaks, one for the liquid phase [see equations (15) and (17)] and one for the gas phase [see equations (16) and (18)]. Each panel represents one spectrum with and without measurement noise, and the background signal.

We simulate spectral data from the detailed model described by equations (1)–(3), to capture the complexity representative of experimental data. Examples of simulated data acquisitions are depicted in Fig. 2 ▸, in this case for four different photon energies, where the spectral spread 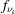 of the photon source has bandwidth 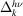 , which depends on the photon frequency (Kachel, 2016 ▸), and 

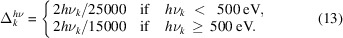

The spatial spread *f*_*r*_ of the photon beam is modeled with a Gaussian function in the plane orthogonal to the propagation direction and is considered parallel all the way to the sample. This simplifying approximation does not account for the fine structure of the photon beam profile such as the granularity caused by speckle noise (Zdora, 2018 ▸). However, the Gaussian approximation is acceptable (Gengenbach *et al.*, 2021 ▸) for simulating data used for validating the applicability of the APE method. Overall, the spatial spread of the photon beam profile is modeled as 

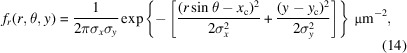

where (*x*_c_, *y*_c_) are the off-center coordinates of the profile (see Fig. 1 ▸) relative to the center of the target. The position (*x*_c_, *y*_c_) determines the greater part of the alignment parameter 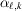 in the photoelectron flux model. The *y*-axis is the cylinder center axis and therefore *y*_c_ does not play a role for modeling the misalignment of the beam and the sample. The origin of the *y*-axis is set by the aperture of the analyzer, point *P* in Fig. 1 ▸. Hence, *y*_c_ represents the distance along the *y*-axis between the maximum of the beam profile and the analyzer aperture *P*. Therefore, *y*_c_ is used to account for the misalignment between the sample and the measurement device and is here set to 70 µm in all simulations. The value of *x*_c_ is varied to simulate different cases of alignment as shown in Fig. 1 ▸. The horizontal spread σ_*x*_ of the photon beam is considerably wider than the vertical spread σ_*y*_. Here, we use values in the same range as the profile described by Fedoseenko *et al.* (2003 ▸), 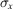 = 100 µm and 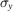 = 25 µm. The photon flux is interpolated with a polynomial function from values obtained during a measurement campaign with characteristic values in the range *F*_*k*_ ∈ [3 × 10^13^, 2 × 10^14^] photon s^−1^.

The channel bandwidth is set to twice the increment 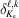 in kinetic energy, *i.e.* 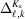 = 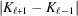 = 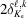 = 0.1 eV. The gain is set to a constant value for all acquisitions, 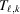 = 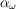 , and the aperture solid angle is assumed to be that of a cone of half angle 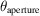 = 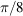 , such that 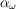 = 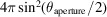 . The integration time 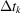 is 10 s for O 1*s* and 60 s for C 1*s*. The spread functions 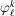 may broaden at higher pass energy and the bandwidth may thereby increase with the photon energy.

Table 1 ▸ gives the values of two energy ratios: (1) the resolution to precision (*i.e.* increment) ratio κ_ana_ and (2) the resolution to cross-section bandwidth ratio κ_σ_. The former, κ_ana_, measures how well the analyzer discretizes its overall spread, *i.e.* how much two neighboring channels overlap. Preferably, two consecutive channels should overlap so that the information is redundant. Values of κ_ana_ smaller than 1 means that there are gaps between two channels and therefore not all information is sampled. Conversely, large κ_ana_ values mean that each channel does not add much information relative to neighboring channels. The value of κ_σ_ indicates how well the device can sample a given peak with bandwidth 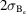 = 0.64 eV. Small absolute values of κ_σ_ mean a good fidelity to the true spectrum and large values mean that the peak will be smeared.

The geometry factor *H*_*P*_(λ_*k*_) is determined, on the one hand, by the photon beam density profile *f*_*r*_ and, on the other hand, by the geometrical parameters of the data acquisition. The attenuation length (Thürmer *et al.*, 2013 ▸) λ_*k*_ of the photoelectrons depends on the kinetic energy range 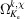 of the photoelectrons, which in turn depends on the photon energy *h*ν_*k*_ and the binding energy of the target (*e.g.* C 1*s*); irradiating the sample at different photon energies is equivalent to varying the attenuation length. For the simulations, we choose photon energy such that the attenuation length λ_*k*_ is in the range [1.3, 3.8] nm (Emfietzoglou & Nikjoo, 2007 ▸). The radius of the cylinder representing the liquid microjet sample is here set to μ_0_ = 10 µm. We assume that the analyzer aperture is far from the LJ in point *P* = ( 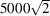 , 0, 5000) µm, where coordinates are relative to the sample center *O* = (0, 0, 0).

We here model two core-level orbitals, C 1*s* and O 1*s*, analogous to the experimental data of Lin *et al.* (2023 ▸) described below. The modes and widths of the O 1*s* and C 1*s* peaks are interpolated from the experimental data by polynomial functions in photon energy; the fits are obtained by means of least-square minimization. The signal from water O 1*s* shows two peaks, one for the liquid phase and one for the vapor phase, and the polynomial model for the modes and spreads are 

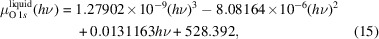




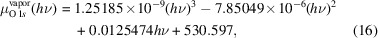




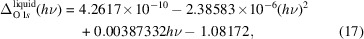




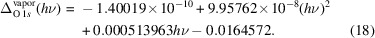

The simulated C 1*s* spectra contain three Gaussian peaks, corresponding to three simulated chemical states of carbon (χ_1_, χ_2_ and χ_3_). These chemical states were inspired by those of carbon in the alkyl chain of sodium dodecyl sulfate (SDS), *i.e.* CH_3_–C, C–CH_2_–C and C–CH_2_–O (Stevie & Donley, 2020 ▸). The polynomial models for the modes 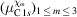 were fitted so that the relative energy shift is interpolated from experimental data. The peak widths 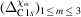 are modeled with linear functions to reflect the peak broadening observed in experimental data (Öhrwall *et al.*, 2015 ▸). However, the absolute positions and widths of the simulated modes do not represent C 1*s* binding energy distributions of the actual carbon chemical states in SDS; instead they were chosen arbitrarily. The polynomial models for the simulated modes and widths are 

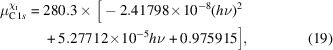




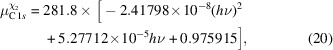




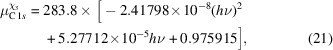




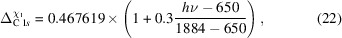




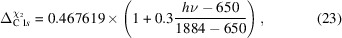




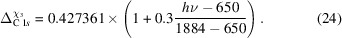

For C 1*s* photionization cross-section density, the relative amplitude of the three peaks are given by the arbitrarily chosen probabilities 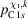 = 0.7 for the main peak and 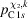 = 0.25 and 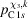 = 0.05 for the secondary peaks. The values 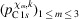 in the simulated spectra do not have a direct physical interpretation and do not reflect expected properties for real SDS samples. The simulated C 1*s* spectra comprise the same number of peaks as expected for actual experimental spectra from SDS, as well as similar background and noise level; however, they differ in terms of peak intensities, modes and widths, as well as the shape of the peaks, which are approximated as Gaussian. In the case of O 1*s*, the relative amplitudes of the gas and liquid peak arise from the distance *x*_c_ along the *x*-axis between the sample and the center of the beam profile. We choose a water vapor concentration inversely proportional to the distance to the center of the sample, 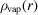 = 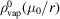 for 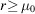 where 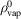 ≃ 0.19 × 10^−3^ mol m^3^ is the water vapor concentration at the sample interface.

The simulations are computed for a target density profile ρ(*r*) that shares similarities with plausible distributions of solutes in aqueous solution (Ottosson *et al.*, 2010 ▸). We choose a smooth edge without surface enhancement,

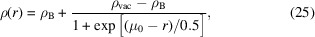

where ρ_B_ [m^−3^] is the concentration of the target species in the sample bulk solution, which is assumed to be uniform throughout the bulk from the center of the sample to a few nanometers below the surface. The vacuum concentration ρ_vac_ [m^−3^] is that of the target species far away from the sample and can be approximated by 0 for non-volatile compounds. Finally, we used a background signal 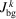 with parametric shape inspired by the experimental data of Lin *et al.* (2023 ▸) and other typical experimental backgrounds (Stevie & Donley, 2020 ▸; Hesse & Denecke, 2011 ▸).

We write the background electron flux as 

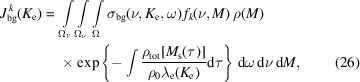

where 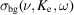 = 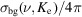 is the background cross section which is the arbitrary term

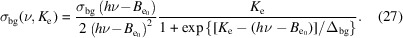




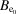
 [eV] is a reference binding energy and Δ_bg_ [eV] is a cut-off width. The threshold 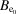 assumes the values 284.0 eV and 547.0 eV for C 1*s* and O 1*s*, respectively, and the cut-off widths Δ_bg_ = 6.0 eV and Δ_bg_ = 3.0 eV. The total background cross section is set to 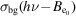 = 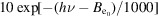 Mb ([10^−22^ m^2^]).

### 3.2. Experimental data

Raw experimental XPS spectra have been obtained from Lin *et al.* (2023 ▸). They studied aqueous solutions comprising organic surfactant SDS (NaC_12_H_25_SO_4_) and inorganic salt sodium chloride (NaCl) at different concentrations and relative mixing ratios. We here focus on spectra for C 1*s*, S 2*p* core-level atomic-like orbitals recorded at different photon energies and their water O 1*s* reference measured at the same kinetic energy to monitor the change in LGPAR. Each XPS spectrum consists of photoelectron signal intensity (count rate) recorded for a range of photoelectron kinetic energies and averaged from 2–22 sweeps to improve the SNR.

## 4. Results

We estimate the alignment parameter 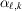 defined in equation (7) for both simulated and experimental data by applying the APE method derived from equation (9). Values for 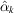 obtained with the APE method are then compared with the estimated alignment from water O 1*s* LGPAR and (for simulated data) with the theoretical values of 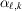 obtained from equation (7).

**Figure 3:**
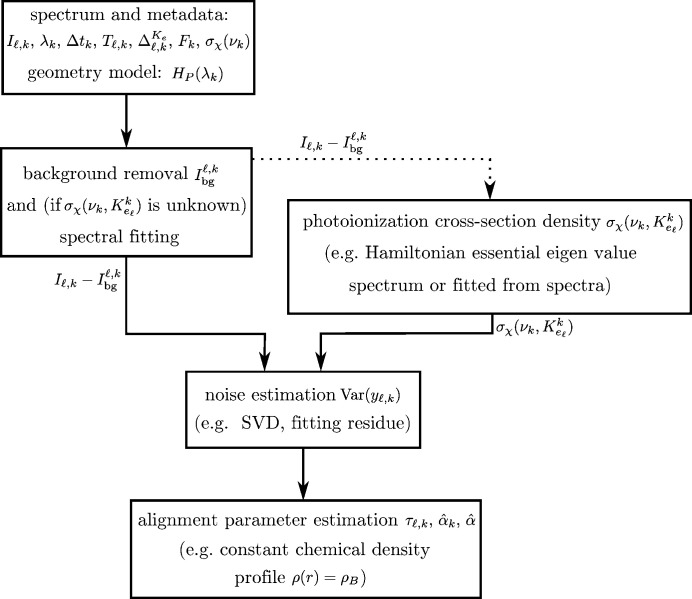
Flow chart for data processing. The solid arrows indicate a necessary dependence while the dashed arrow indicates an optional dependence.

The flow chart in Fig. 3 ▸ shows the logical flow from the data to the output of the APE method. In both simulated and experimental cases, the first processing step is background removal and peak fitting, here performed with the *SPANCF* package. The following step depends on knowledge of the photoionization cross-section density 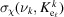 – either it is known from theory, *e.g.* from density functional theory simulations (Toffoli *et al.*, 2007 ▸), or it must be estimated from the spectral data, *e.g.* fitted peaks. Subsequently, the noise is estimated, either as the residue of the fits or from a model-based method (see Appendix *A* [App appa]), after which the alignment parameter 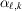 can be estimated from equation (9).

In the following, we show an example of noise estimation for simulated data and applications of the APE method for simulated (Section 4.1) and experimental (Section 4.2) data.

### 4.1. Simulated data

The simulated O 1*s* and C 1*s* spectra are described in Section 3.1 and used as a proof of concept for the APE method. For the simulated C 1*s* spectra, the number of peaks is known at the time of fitting, but the mode of the peaks and their widths, as well as the background, are kept unknown *a priori*. The O 1*s* peak fitting and background removal is processed assuming one peak for each phase not knowing the modes and width as well.

#### 4.1.1. Noise estimation

We estimate the noise in the spectra following the two possible paths shown in Fig. 3 ▸ to illustrate the different possibilities. Either the noise is directly estimated as the residue from the *SPANCF* fits or it is indirectly computed using the *SPANCF* fits for the photoionization cross-section density model and then used as an input to a singular value decomposition (SVD) based method (see Appendix *A* [App appa]). Other noise estimation methods and fitting methods can be deployed, but are not investigated here.

**Figure 4:**
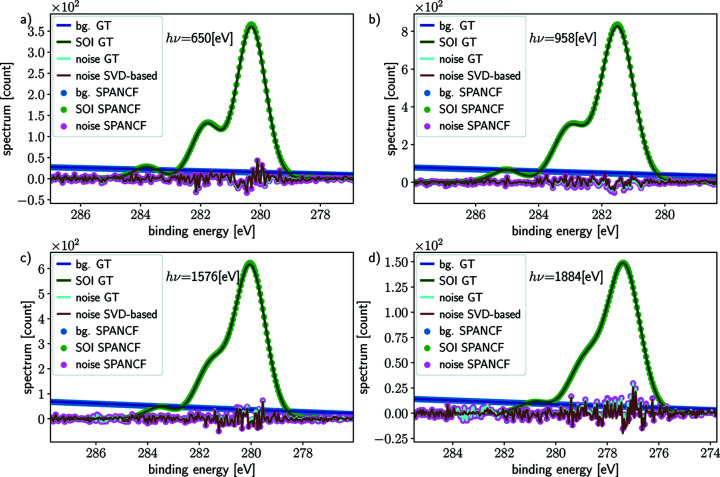
Peak fitting, noise and background removal for C 1*s* simulated data for four photon energies *h*ν ∈ {650, 958, 1576, 1884} eV. The true signal of interest (green) and the background (blue) are compared with the estimation computed with *SPANCF* routines (scattered dots). The true noise (brown) is similar to the estimated noise, either from *SPANCF* processing (pink) or the proposed noise estimation (purple) described in Appendix *A* [App appa].

The results for C 1*s* simulations are shown in Fig. 4 ▸ for one horizontal off-center distance 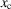 = 100 µm for illustration. In each panel of Fig. 4 ▸, the same quantities are plotted for four photon energies *h*ν_*j*_, (*a*) 650 eV, (*b*) 958 eV, (*c*) 1576 eV and (*d*) 1884 eV, corresponding to different attenuation lengths (Emfietzoglou & Nikjoo, 2007 ▸) of emitted photoelectrons in the sample (*a*) 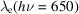 = 1.35 nm, (*b*) 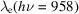 = 1.96 nm, (*c*) 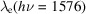 = 3.19 nm and (*d*) 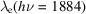 = 3.80 nm. The blue scatter dots in each panel of Fig. 4 ▸ depict the background extracted from the acquired spectrum and are in each case almost identical to the true background (solid dark blue line). The fits are performed on the spectrum averaged over repeated (simulated) acquisitions, with more sweeps providing better SNR of the average spectrum. The fitted signal of interest (SOI) represented with green scatter dots is also similar to the true SOI (dark green solid line), even when the SNR for each spectrum is low. This is due to the large number (50 for the cases shown) of sweeps allowed by the simulations. From the fits and the estimated background, the noise is estimated as the residue (pink scatter dots) and compared with the true (simulated) noise (cyan solid line). The brown solid line represents the noise estimated from the SVD-based method and is virtually indistinguishable from the true (simulated) noise (cyan solid line). These results illustrate that the SVD-based method and the fit residue can effectively estimate the noise from the spectra. For the SVD-based method, this is explained by the very low rank (rank 1) of our discretized model, whereby all the signal of interest is encoded in one left singular vector, while the rest of the vectors encode the noise.

#### 4.1.2. Alignment parameter estimation

**Figure 5:**
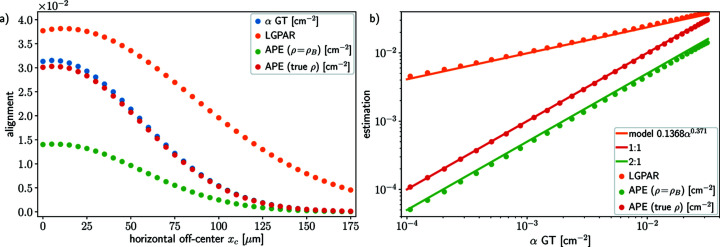
Comparison of the LGPAR [a.u.] (orange), true alignment parameter 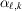 , equation (7), in cm^−2^ (blue scatter dot), and alignment parameter estimation (APE), equation (9), in cm^−2^ (green and red), for simulated data. The red APE is computed using the true density profile and the green APE with a constant density profile. (*a*) The four quantities are plotted against 36 horizontal off-center distances *x*_c_ ∈ [0, 175] µm. (*b*) The LGPAR and the estimated alignment parameter are plotted against the true parameter. The orange solid line represents the fitted model, and the red and green solid lines are, respectively, the 1:1 and 2:1 curves to guide the eye. The estimated 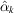 and true alignment parameter 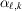 are linearly correlated and the LGPAR exhibits a non-linear correlation with the true alignment parameter α.

The O 1*s* XPS data were simulated for 36 different horizontal off-center distances *x*_c_ uniformly spaced in the interval [0, 175] µm. For each simulated spectrum, we determine (i) the true value of the alignment parameter 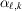 , referred to as the ground truth (GT), according to the definition in equation (7), (ii) the estimation of 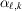 using the approximation method (APE) derived from equation (9), and (iii) the conventional experimental proxy given by the water O 1*s* liquid to gas peak ratio. These three alignment estimates are shown in Fig. 5 ▸(*a*), as functions of the horizontal off-center distance *x*_c_. Also shown are the true alignment parameter 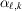 (blue), the APE estimates, using the true profile ρ(*r*) (red), and using a constant profile ρ(*r*) = ρ_B_ (green), and the LGPAR (orange). For each curve, the maximum does not occur at the center of the sample (*x* = 0) but rather at the distance *x*_max_ close to point *R* in Fig. 1 ▸, where the analyzer points to the sample.

Fig. 5 ▸(*b*) shows the APE using the variance of the noise and a constant density profile ρ(*r*) = ρ_B_ (green scatter dots) and using the same concentration profile as for the simulation (true profile, red scatter dots), respectively, and the water O 1*s* LGPAR proxy (orange scatter dots) against the GT (*x*-axis). The agreement between the APE estimations using the true target profile and the true values of the alignment parameter 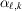 supports the use of the APE method. The APE using constant density profile ρ(*r*) = ρ_B_ underestimates the alignment parameter by slightly more than a factor of two, but the correlation is linear and positive. The main reason for this difference is the use of a constant concentration profile equal to the bulk concentration for positions where the concentration is significantly different. We opt to keep this profile in our comparison because, *a priori*, the concentration profile of the target in the sample is not known and a constant profile, corresponding to a homogeneous (isotropic) distribution throughout the sample, is a reasonable first guess for many target species. The water O 1*s* LGPAR is also clearly correlated with the true alignment parameter 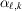 , but not linearly. Instead, the power law 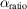 = 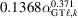 can here be used to describe the relation between the water O 1*s* LGPAR and the GT in Fig. 5 ▸(*b*).

**Figure 6:**
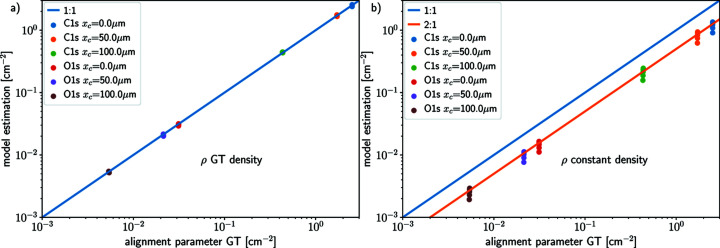
Comparison between the true alignment parameter 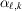 and the APE 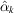 for a set of simulated data processed using the *IGOR* routine for background removal and peak fitting. Panel (*a*) shows the APE 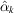 using the true density profile and panel (*b*) shows the APE 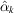 using a constant density profile (bulk concentration).

In Fig. 6 ▸, the APE computed for both processed O 1*s* and C 1*s* XPS spectra with off-center distances *x*_c_ ∈ {0, 50, 100} µm are plotted against the true alignment parameter 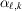 [given by equation (7)] for two cases: panel (*a*) using the true density profile ρ(*r*) and panel (*b*) using a constant density profile with the bulk concentration value ρ(*r*) = ρ_B_. In both cases the correlation between the true alignment parameter 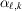 and the APE 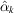 is linear. The spread observed in APE in the case of a constant density profile ρ(*r*) = ρ_B_ is due to this approximation of the density, resulting in an overestimation of the contributions to the photoelectron signal from the target species near the surface and exacerbating the spread of the alignment. The spread in the true alignment parameter 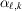 due to the different probed photon energies and attenuation lengths is finite, but in this case negligible.

### 4.2. Experimental data

Experimental XPS spectra were fitted following the same methods used for simulated data and mentioned in Section 2. The background was approximated with a straight line. The asymmetry of averaged peaks, caused by small shifts of peak positions between consecutive sweeps, was neglected.

**Figure 7:**
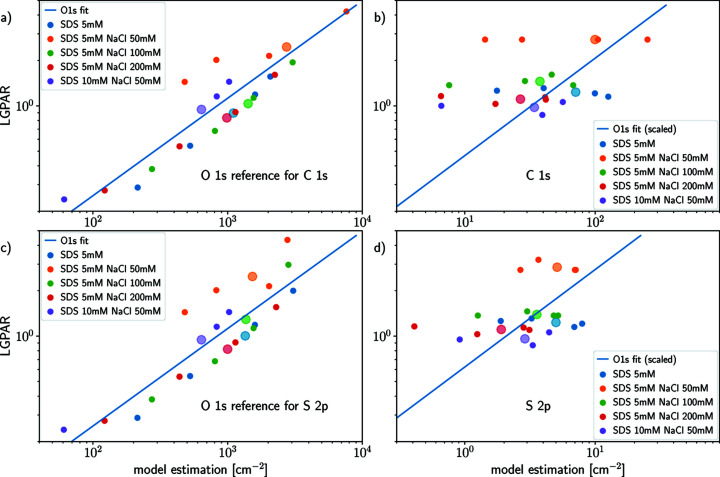
Comparison of the LGPAR and 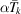 from the APE for experimental data. Three cases are considered, (*a*) and (*c*) O 1*s*, (*b*) C 1*s* and (*d*) S 2*p*. The different colors for the scattered dots correspond to the different experimental conditions. For an experimental condition, each scatter dot represents a different photon energy. For each condition the average 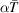 is represented by a larger and lighter color dot. The blue line is the linear fit (in log–log space) for the O 1*s* data, and an offset has been applied to the fit for C 1*s* and S 2*p* for the sake of comparison.

From the experimental data, the water O 1*s* LGPAR was estimated using the fitted curves obtained with *SPANCF*. Using the known values for the photon flux *F*_*k*_, atomic subshell total photoionization cross section σ_χ_(ν_*k*_), the kinetic energy step 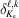 and the integration time Δ*t*_*k*_, the remaining multiplicative factors, *i.e.* 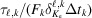 = 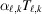 , were estimated using equation (9). From the observation that the AP, all things being equal, is constant, we plot the two quantities 

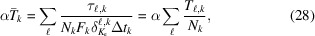




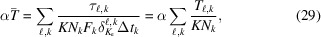

against the water O 1*s* LGPAR. The results are shown in Fig. 7 ▸: binary 5 m*M* SDS (blue scattered dots), ternary solutions of 5 m*M* SDS and 50 m*M* NaCl (orange scattered dots), 5 m*M* SDS and 100 m*M* SDS (green scattered dots) and 5 m*M* SDS and 200 m*M* NaCl (red scattered dots), respectively. Ternary 10 m*M* SDS and 50 m*M* NaCl solution is shown in violet.

For each dataset, *i.e.* for each given solution with composition (SDS [m*M*], NaCl [m*M*]), the collection of pairs ( 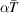 , water O 1*s* LGPAR) designating the average of the estimated product 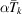 and the water O 1*s* LGPAR for each target, are represented by a larger dot with a lighter shade of color. The blue curve has been fitted from the O 1*s* data as a linear function in log–log space. To facilitate their comparison, offset curves are shown for the C 1*s* and S 2*p* data. The agreement between the estimated product 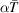 and the water O 1*s* LGPAR proxy for the O 1*s* data is clear: no significant outliers perturb the fit. The discrepancies between the linear fits [least square in Figs. 7(*a*) and 7(*c*), blue lines] and the data pairs ( 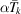 , water O 1*s* LGPAR) can be explained by the multiple steps for data processing, the modeling errors and the assumptions regarding the integrating time and the intensity response function.

For C 1*s* and S 2*p* data, we use the ratio with respect to the O 1*s* reference with the closest kinetic energy to estimate the water O 1*s* LGPAR alignment proxy. Here, the agreement between the product 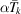 and water O 1*s* LGPAR alignment proxy is not as strong as for O 1*s*; however, the average 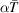 and the water O 1*s* LGPAR (large dots with light shade) are linearly correlated in log–log space.

The assumption that the water O 1*s* LGPAR can be used for the C 1*s* and S 2*p* peaks does not seem to hold for each individual acquisition.

## 5. Discussion and conclusion

We have presented a method for estimating the alignment parameter in the framework of synchrotron-radiation-excited surface-sensitive XPS experiments and applied it for a series of XPS measurements carried out on liquid microjet samples. The requirements for applying the method are:

(i) Availability of the raw spectral data (photoelectron counts as a function of kinetic energy) without processing such as smoothing or noise removal.

(ii) Obtaining the spectral background and collection of fitted peaks attributed to distinct chemical species.

(iii) Metadata pertaining to the experimental configuration (attenuation length of the photoelectrons in the sample and sample geometry and dimensions).

Application of the method was illustrated for simulated and experimental XPS spectral data obtained from cylindrical LJ samples. However, the method can be readily applied to other sample geometries, such as planar or spherical (droplet) samples. The method enables any combination of unknown experimental parameters in the multiplicative factor of equation (5) to be retrieved, *e.g.* the alignment 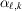 and the intensity response functions 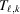 .

The APE is implemented in the *PROPHESY* suite of *Julia* packages (Ozon *et al.*, 2023 ▸) which is open and freely available. As long as an XPS experiment produces the metadata corresponding to the measurement model and the raw spectra, the method is readily deployable without further mathematical considerations. The background and spectral fits may be provided from another XPS data analysis framework (*e.g.* *SPANCF*) or computed from the *PROPHESY* framework which implements a background-removal algorithm and a model-free spectrum estimation method. The spectral peaks do not need to be fitted for the method to work with a series of individual peaks – only the overall spectrum needs to be fitted; however, the results from a peak fitting method are readily applicable for the APE method. Hence, it is not required to know the number of peak or chemical states for a target orbital, nor is it necessary to assume a model for the peaks (*e.g.* Gaussian profile). The computation of the estimates τ_ℓ,*k*_, 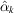 and 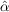 can be executed online; the extra computational time for these estimates is negligible compared with the estimation of the background and the spectral fits.

Our results show that the water O 1*s* LGPAR can be used as a proxy for the alignment parameter 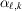 , but the evidence is not as strong in the case of C 1*s* and S 2*p* as in the case of O 1*s*. The discrepancies in the geometry model, background removal and peak fitting can partially explain the errors between the O 1*s* ( 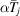 , LGPAR) fits and the data [Figs. 7 ▸(*a*) and 7(*c*)]. However, for C 1*s* and S 2*p*, the deviations between ( 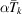 , LGPAR) fits and the data [Figs. 7 ▸(*b*) and 7(*d*)] are too significant to be explained only by modeling error. Potentially, the experimental data quality for C 1*s* and S 2*p* could partly explain the deviation. We therefore do not encourage using the O 1*s* LGPAR for target orbitals C 1*s* and S 2*p*, even if the mean values of LGPAR and 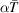 correlate well.

The estimators devised for the APE, τ_ℓ,*k*_, 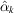 and 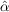 produce satisfactory estimates; however, they suffer limitations. For instance, the noise in the data directly affects the estimates of τ_ℓ,*k*_ which affect the estimator 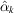 ; however, it is averaged out over the number of measurements in a spectrum. The bias due to the noise in the data is 0-mean, hence these estimators are unbiased with respect to the measurement noise as long as the noise model holds. The methodology relies on the noise model and, therefore, if the noise deviates considerably from a Poisson distribution or if the expected measurement shows a significant offset due to dark current of reading noise, the method may require modifications to be applied. For instance, for photoelectron counting devices other than CCD or CEM, the noise model should be modified accordingly.

The estimators τ_ℓ,*k*_, 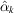 and 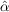 rely either on simulation or estimators of the background and peaks which can be biased. These biases potentially represent an important source of error in the estimation of τ_ℓ,*k*_. Contrary to the measurement noise, the background estimator bias is not averaged out in the estimator 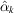 ; rather, it accumulates. When the photoionization cross-section density is estimated from curve fitting, the bias is present in the numerator and denominator of the estimator of τ_ℓ,*k*_ which reduces the overall bias.

The error in the concentration profile ρ is illustrated in Fig. 6 ▸(*b*). The spread in the estimates 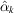 is overwhelmingly originating from the error in the concentration profile. The contribution due to the spectrometer and light approximations are effectively negligible. Similarly to the error in the concentration profile, the error in the geometry, *i.e.* approximation of 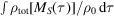 , by the distance function *d*_*P*_(*M*), completely omits the photoelectric signal from low-density liquid and vapor at the edge of the sample. However, the data simulation was carried out including the signal emerging from the surrounding vapor, while the estimations were made with the simple sharp volume model, which does not account for the vapor.

In Fig. 6 ▸, the spread in the true alignment parameter α_ℓ,*k*_ for each misalignment value *x*_c_ and orbital is negligible, supporting the claim that for a given target orbital the AP should be a constant of the experiment across the photon energies. However, if the true value of the AP is almost independent of the attenuation length, the estimate depends on the attenuation length value which is known only with limited precision. Further numerical experiments (not shown in this work) conducted with erroneous attenuation length values show that the AP estimates depend linearly on the attenuation length. For instance, an AP estimation computed with an attenuation length value up to 2.8 times greater than that used for the simulation leads to an estimated AP value that differs from the true value with the same factor. This is the same order of magnitude of error as that due to the concentration profile approximation.

For a given setup in Fig. 7 ▸, the variability can also be attributed to the assumption that the intensity response function *T*_*k*_ is constant across probed attenuation lengths. However, the average estimates 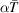 suffer less from these assumptions because it estimates the average spectrometer characteristic over the photon energy.

With this method, we can estimate the alignment parameter used in simplified XPS measurement models. This eliminates the need for using relative peak areas for compensating for missing parameters such as the alignment parameter, the intensity response function 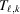 , the photon flux *F*_*k*_, and the total photoionization cross section 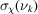 . In the LJ framework, we recommend computing the LGPAR and estimate the alignment parameter for each experiment using XPS spectra obtained at different photon energies.

## Figures and Tables

**Table 1:** Relative spread of the kinetic energy spectrum for three photon energies *h*ν_*k*_ [eV] The analyzer has resolution 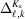 = 0.1 eV and precision 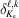 = 0.05 eV – the distance between channels. The analyzer ratio 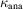 = 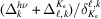 and the cross section to analyzer ratio 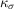 = 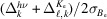 indicates the quality of sampling.

Photon energy	Energy spread 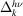	κ_ana_	κ_σ_
375	0.015	2.3	9%
500	[0.02, 0.03]	[2.4, 2.7]	∼10%
1500	0.1	4	15%

## Appendix A Noise estimation

One of the challenges related to analyzing and interpreting XPS data is the estimation and removal of the noise in the recorded spectra. Rather than manual estimation based on the observation of each data set, we implement a simple method based on the discretized model and its SVD (Stewart, 1993 ▸). Assuming that the background signal has been removed, then the remaining signal is

where τ_*k*_ is a vector with entries τ_ℓ,*k*_,  is a vector with entries  , and Γ_*k*_ is a diagonal matrix representing the variance of the measurement noise. We write  as the SVD  , for which the columns of the orthogonal matrix *U*_*k*_ form a base for the vector *I*_*k*_, *S*_*k*_ is a diagonal matrix with the singular value entries *s*_*i*_, and *V*_*k*_ is an orthogonal basis for the concentration profile ρ of the target with respect to the sample surface. Projecting the measured signal on the basis vectors of the left space,

and observing that the signal  is concentrated where the singular values are significant, *e.g.*    with *s*_th_ a given threshold,  ≃  with  comprising the *i*_0_ − 1 most significant singular values, where *i*_0_ is the first *i* such that    . Hence, for a photoelectron spectrum consisting of *N*_*k*_ measurement points, we use only the *N*_*k*_ − *i*_0_ + 1 vectors in *U*_*k*_ corresponding to the smallest singular values,

so that we obtain the approximation

which is the projection of the noise on the basis  . Since  is an outer product, only one singular value is significantly non-zero, such that *i*_0_ = 2. An estimation of the noise is then given using the null space basis as
